# Serum *N*-Glycans as Independent Predictors of Death: A Prospective Investigation in the AEGIS Cohort

**DOI:** 10.1016/j.mcpro.2025.101217

**Published:** 2025-10-15

**Authors:** Iago Carballo, Óscar Lado-Baleato, Manuela Alonso-Sampedro, Róisín O’Flaherty, Radka Saldova, Francisco Gude, Arturo González-Quintela

**Affiliations:** 1 Department of Internal Medicine, University Hospital Complex of Santiago (CHUS), Santiago de Compostela, Spain; 2 Research Methods Group (RESMET), Health Research Institute of Santiago de Compostela (IDIS), Santiago de Compostela, Spain; 3 Network for Research on Chronicity, Primary Care, and Health Promotion (RICAPPS-ISCIII), Health Research Institute of Santiago de Compostela (IDIS), Santiago de Compostela, Spain; 4 ISCIII Support Platforms for Clinical Research, Health Research Institute of Santiago de Compostela (IDIS), Santiago de Compostela, Spain; 5 Department of Chemistry, Maynooth University, Maynooth, Co. Kildare, Ireland; 6 Kathleen Lonsdale Institute for Human Health Research, Maynooth University, Maynooth, Co. Kildare, Ireland; 7 GlycoScience Group, National Institute for Bioprocessing Research and Training, Dublin, Ireland; 8 UCD School of Medicine, College of Health and Agricultural Science (CHAS), University College Dublin (UCD), Dublin, Ireland; 9 Department of Psychiatry, Radiology, Public Health, Nursing, and Medicine, University of Santiago de Compostela (USC), Santiago de Compostela, Spain

**Keywords:** biomarkers, cancer, cardiovascular disease, death, glycomics, mortality, *N*-glycome

## Abstract

Total *N*-glycome in blood serum or plasma provides information about all serum/plasma protein enzymatic glycosylation, a tightly regulated cotranslational and post-translational modification. Total plasma/serum *N*-glycome has shown specific patterns (signatures) in patients with high-mortality pathologies, such as cancer and cardiovascular diseases; thus, we explored the capacity of total serum *N*-glycome to predict mortality in a general adult population. This prospective cohort study was performed in a municipality in Spain including a random sample of 1516 adults. Participants were profiled for total serum *N*-glycome at baseline. Serum enzymatic *N*-glycan release was performed on a robotic platform followed by hydrophilic interaction chromatography–ultraperformance liquid chromatography glycan separation. The computerized medical records were checked at a median follow-up of 7.52 years to collect the date and cause of all deaths. *N*-glycan groups from total serum were used to develop mortality prediction models. Total serum *N*-glycome peak (GP) 16, mainly composed of A2[3]BG1S[3]1, predisposed to all-cause mortality; GP 22, mainly composed of FA2G2S[6]1, protected from all-cause mortality. The balance between them predicted all-cause mortality incidence over time (area under the curve [AUC], 0.810 [0.773–0.847]). Similar results were obtained for cancer mortality, with GPs 16, 17, 22, and 23 (AUC, 0.786 [0.728–0.843]); and for cardiovascular mortality, with GPs 7 and 9 (AUC, 0.747 [0.645–0.850]). Their predictive powers had an independent and additive effect on classical prediction factors. The balances between specific GPs are independent predictors of all-cause, cancer, and cardiovascular mortality and could contribute significantly to improving prognostic tools.

## Highlights

• Total serum *N*-glycans predict the incidence of death in an adult population sample.
• *N*-glycome peak (GP) 16, mainly composed of A2[3]BG1S[3]1, predisposes to death.
• GP 22, mainly composed of FA2G2S[6]1, protects against death.
• GP16 and 22 have independent and additive effect on classical prediction factors.
• GP16 and 22 may contribute to improve classical prediction tools.

## Graphical Abstract

**Figure:**
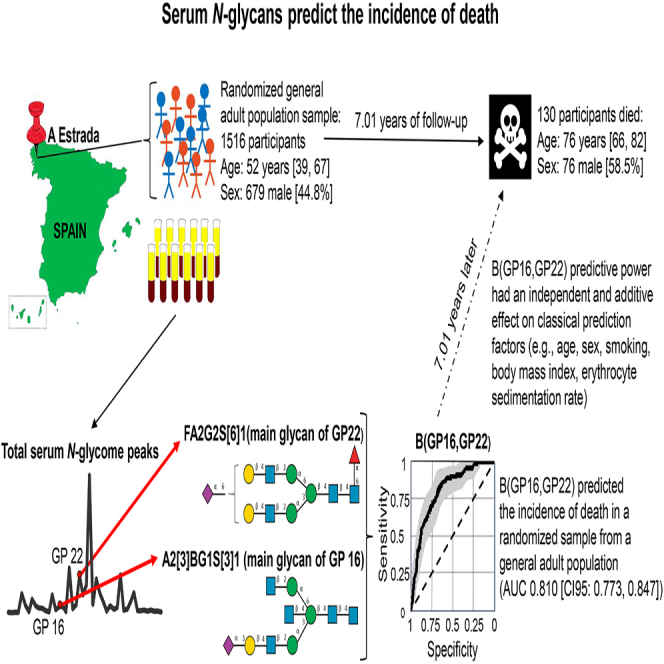

## In Brief

Total serum *N*-glycome encompasses all glycans linked to serum proteins through enzymatic glycosylation, a tightly regulated cotranslational and post-translational modification. Total plasma *N*-glycome has shown specific patterns (signatures) in patients with high mortality pathologies, such as cancer and cardiovascular diseases. This study is the first to investigate total serum *N-*glycome to predict mortality in a general adult population. Furthermore, it reveals the strong predictive power of total serum *N-*glycome to predict all-cause mortality, cancer mortality, and cardiovascular mortality.

Glycans are simple or complex molecules that play a critical structural and metabolic role in a cell or an organism. Glycation is a nonenzymatic reaction that leads to the binding of a glycan to a protein, lipid, or another glycan. However, glycosylation is a sophisticated biological process regulated by many genes, transcription factors, and signaling pathways that takes part in cotranslational and post-translational modifications. The comprehensive study of all glycan structures, generation, modification, degradation, regulation, and function of living beings is known as “glycomics” (1).

At least half of all human proteins are glycoproteins, and most of them are covalently linked to the amide nitrogen of an asparagine that belongs to an asparagine–X–serine/threonine consensus sequence of a protein, in which X can be any amino acid except proline (1, 2). Total plasma/serum *N*-glycome encompasses all glycans linked to plasma/serum proteins through this type of bonding, and their analysis provides information about patterns of glycation (signatures) that are common in various diseases.

Total plasma/serum *N*-glycome has shown an increased abundance of triantennary and tetra-antennary *N*-glycans with higher sialylation and fucosylation in patients with various types of cancer, such as colorectal (3), ovarian (4), and pancreatic (5). Furthermore, a fucosylated glycoform of α-fetoprotein (the *Lens culinaris* agglutinin–reactive α-fetoprotein) in serum had been shown to support hepatocellular carcinoma diagnosis (6) and was approved by the Food and Drug Administration as a biomarker for its early detection. In patients with prevalent and incident cardiovascular disease, total plasma *N*-glycome has shown increased α2,6-sialylation and decreased α2,3-sialylation, as well as increased bisection, decreased fucosylation, and decreased galactosylation of immunoglobulin G-related glycans (7). Moreover, macrovascular complications in patients with type 2 diabetes mellitus were found to be related to decreased levels of FA2[6]G1 (8), and a glycan-based prediction model of incident cardiovascular events was also considered (9). To the best of our knowledge, however, total serum *N*-glycome (TSNG) has not been studied in cardiovascular, cancer, or all-cause mortality in depth.

Mortality is the classical primary endpoint in clinical research, and cardiovascular diseases and cancer remain the leading causes of death globally (10, 11). However, there are no population-based predictive models capable of accurately identifying individuals at high risk of mortality from cardiovascular diseases or cancer. Moreover, population screening programs are currently available for only a limited number of cancer types (12), and more than half of the cardiovascular events occur in adults not classified as high risk according to traditional risk factor assessments (13). On the other hand, all-cause mortality is influenced by numerous factors, each with a generally low relative weight, so it is more difficult to assess and interpret than specific causes of death in research studies. Nevertheless, all-cause mortality is considered a purer endpoint than disease-specific mortality, as it helps mitigate issues, such as bias in patient selection, missing data, and changes in classification over time (14).

The aim of this study was to identify predictive biomarkers of all-cause, cancer, and cardiovascular mortality in a general adult population and to explore its usefulness in the general adult population as a prognostic tool alone and in combination with age, sex, and other common risk variables.

## Experimental Procedures

### Study Design and Data Sources

The A-Estrada Glycation and Inflammation Study (AEGIS) is a prospective cohort study developed in A-Estrada, a municipality in Northwestern Spain. An outline of the study is available at www.clinicaltrials.gov (code: NCT01796184). A-Estrada had an adult population (aged older than 18 years) of 18,474 when the study began in 2012. An age-stratified random sample of 3500 adults was drawn from Spain’s National Health System Registry, which covers more than 95% of the population. From the initial 3500 individuals, 2230 could be assessed for eligibility and displayed no exclusion criteria (no health care, moved away, died, and no response to communication attempts); of these, 1516 agreed to participate (overall participation rate, 68%). Participation was higher among women than men (71% *versus* 65%, respectively). There were no significant differences in terms of age or residence (rural *versus* urban) between participants and nonparticipants.

From November 2012 through March 2015, all participants were successively contacted and asked to attend the primary care center for evaluation through a clinical interview that included a structured questionnaire, physical examination, and fasting venous blood sampling (n = 1516, 44.7% men, aged 18–91 years; median, 52 years). Most recently, a physician examined the computerized medical records of all participants up to February 2023, which included all information about primary and hospital medical care provided by the Spanish National Health System. Participants with a status of death in the medical records were noted, with the date and cause of death collected.

### Ethical Issues

All participants provided written informed consent. The general survey was approved by the Galician Regional Ethics Committee (code: 2010-315) and conformed to the current Helsinki Declaration.

### Assessment of Overweight and Obesity

Body mass index (BMI) was calculated as weight (in kilograms) divided by the square of height (in meters). Participants were classified according to BMI as normal weight (<25 kg/m^2^), overweight (25–30 kg/m^2^), or obese (>30 kg/m^2^).

### Assessment of Metabolic Abnormalities

Participants were considered as having metabolic syndrome if they had at least three of the following Adult Treatment Panel III criteria (15): (1) abdominal obesity; (2) hypertriglyceridemia; (3) low levels of high-density lipoprotein (HDL) cholesterol; (4) increased blood pressure; and (5) hyperglycemia.

### Assessment of Smoking and Alcohol Consumption

Consumers of at least one cigarette per day were classified as smokers. Participants who had quit smoking during the preceding year were still considered smokers.

Alcohol consumption was evaluated in standard drinking units (16) by summing the number of glasses of wine (1 unit, ∼10 g), bottles of beer (1 unit, ∼10 g), and spirits (2 units, ∼20 g) regularly consumed per week. Participants with an alcohol consumption of 1 to 13 units/week were considered as light drinkers, those with 14 to 27 units/week as moderate drinkers, and those with ≥28 units/week as heavy drinkers.

### Assessment of Physical Activity

All study participants completed the short version of the International Physical Activity Questionnaire (available at https://sites.google.com/view/ipaq/home), which has been validated in Spain (17). The questionnaire allows for the calculation of metabolic equivalents of various tasks and for stratification of habitual physical activity as low, moderate, or high (18).

### Assessment of Glomerular Filtration Rate

The glomerular filtration rate (GFR) was estimated by the 4-variable Modification of Diet in Renal Disease equation, using standardized serum creatinine values measured with isotope dilution mass spectrometry–traceable assays (4-variable Modification of Diet in Renal Disease–isotope dilution mass spectrometry equation): estimated GFR = 175 × (standardized creatinine)^−1.154^ × (age)^−0.203^ × 1.212 (if black) × 0.742 (if female) (19).

### Determination of Fasting Plasma Glucose

Fasting plasma glucose (FPG) levels were determined in fresh serum samples from fasting participants by the glucose oxidase method in an ADVIA 2400 Clinical Chemistry System (Siemens).

### Determination of Glycated Hemoglobin

Glycated hemoglobin (HbA1c) levels were determined in fresh serum samples by high-performance liquid chromatography in an ADAMS A1c HA-8160 analyzer (ARKRAY); all HbA1c values were converted to Diabetes Control and Complications Trial–aligned values.

### Cholesterol Assay

Cholesterol levels were determined in fresh serum samples from fasting participants by the enzymatic method in an ADVIA 2400 Clinical Chemistry System.

### Low-Density Lipoprotein Cholesterol and HDL Cholesterol Assays

Low-density lipoprotein cholesterol and HDL cholesterol levels were determined in fresh serum samples from fasting participants by the elimination/catalase method in an ADVIA 2400 Clinical Chemistry System.

### Aspartate Aminotransferase Assay

Aspartate aminotransferase levels were determined in fresh serum samples from fasting participants by the International Federation of Clinical Chemistry–modified method in an ADVIA 2400 Clinical Chemistry System.

### C-Reactive Protein Assay

Wide-range C-reactive protein (CRP) concentrations were measured in fresh serum samples using commercial latex-enhanced immunoturbidimetry in an ADVIA 2400 Clinical Chemistry System.

### Erythrocyte Sedimentation Rate Assay

The erythrocyte sedimentation rate (ESR) was measured in blood drawn in vacuum tubes containing K3EDTA (Becton Dickinson) employing an automated TEST-1 device (Alifax).

### Interleukin-6 Assay

Interleukin-6 concentrations were measured in fresh serum samples using a commercial chemiluminescent immunoassay in an IMMULITE 2000 System (Siemens).

### Tumor Necrosis Factor-Alpha Assay

Tumor necrosis factor (TNF)-alpha concentrations were measured in fresh serum samples using a commercial chemiluminescent immunoassay in an IMMULITE 2000 System (Siemens).

### Serum *N*-Glycan Analyses

The complete procedure developed by the authors has recently been published (20). *N*-glycans were profiled by a modified high-throughput automated method from 5 μl of serum samples that had previously been stored at −80 °C for further use (21). Briefly, the samples were denatured, and *N*-glycans were enzymatically released from the protein backbone *via* peptide:*N*-glycosidase F. The glycans were then immobilized on solid-supported hydrazide beads, and excess reagents were removed by centrifuge filtration. The glycans were released from the solid support and labeled with fluorophore 2-aminobenzamide.

Hydrophilic interaction chromatography–ultraperformance liquid chromatography was performed, assigning glucose unit values from retention times. The chromatograms were all separated in the same manner into 46 peaks according to Saldova *et al.* (22), and the amount of glycans in each peak was expressed as a percentage of the total integrated area. Glycan structures were annotated using the symbol nomenclature for glycans and DrawGlycan-SNFG software (23, 24), with the assistance of GlycoStore.org (accessed on November 11, 2021; currently available at www.glycosmos.org/glycostore/uplc) (25).

A summary of glycome peaks (GPs) and the corresponding main *N*-glycan structures are shown in the recent article describing the complete procedure (20). Groups of GPs were defined from their common features, as follows (22):

Sialylation: S0 (neutral, GP1−15); S1 (monosialylated, GP16−23 + GP30); S2 (disialylated, GP24−29 + 31); S3 (trisialylated, GP32−40); and S4 (tetrasialylated, GP41−46).

Galactosylation: G0 (agalactosylated, GP1−2 + GP4−5 + GP6/2 + GP12/2); G1 (monogalactosylated, GP3 + GP7−10 + GP12/2 + GP16−18 + GP21/2); G2 (digalactosylated, GP13−15 + GP19−20 + GP21/2 + GP22−28); G3 (trigalactosylated, GP29 + GP31−37); and G4 (tetragalactosylated, GP30 + GP38−46).

Branching: A1 (monoantennary, GP1−3 + GP12/2 + GP21/2); A2 (biantennary, GP4−5 + GP6/2 + GP7−10 + GP12/2 + GP13−20 + GP21/2 + GP22−28); A3 (triantennary, GP29 + GP31−37); and A4 (tetra-antennary, GP30 + GP38−46).

Oligomannose: GP6/2 + GP11.

Fucosylation: Core-fucose (GP2 + GP5 + GP6/2 + GP8−10 + GP14−15 + GP17−18 + GP22−23 + GP27−28 + GP36 + GP44/2) and outer-arm fucose (GP37 + GP40 + GP41/3 + GP45 + GP46/3).

In addition, mass spectrometry–assisted glycan characterization was performed for two representative samples and a technical replicate (20). Otherwise, the major glycans were identified and assigned based on their glucose unit values crossreferenced in GlycoBase, later migrated to GlycoStore and now to GlyCosmos, and based on previous assignments in the study by Saldova *et al.* (22, 26).

A summary of *N*-glycan structures identified by mass spectrometry and their correlation with *N*-glycan structures identified by hydrophilic interaction chromatography–ultraperformance liquid chromatography is shown in a recent article describing the complete method (20).

### Statistical Analysis

Mann–Whitney and Chi-squared tests were applied to check for differences in continuous and categorical variables, respectively, in the participant groups.

Survival time was defined from the date of enrollment in AEGIS to the date of exitus. Censored time was considered for participants who were alive at the end of the study or were lost during follow-up. Cumulative incidence curves were obtained from the inverse of the Kaplan–Meier estimator, and differences in survival rates between patient groups were tested using the log-rank test.

An automatic variable selection algorithm based on ridge regression techniques was used to establish main risk factors of death. Specifically, an elastic net regularization method was fitted, where the choice of the optimum lambda parameter was based on the model C index obtained from 10 crossvalidations. Selected variables, with *p* values higher than 0.05 in a multivariate Cox regression model, were discarded from the final model. Moreover, nonlinear effects of continuous variables were estimated through spline functions. Results were expressed as hazard ratios.

*N*-glycome data were analyzed following up-to-date compositional data analysis techniques. The exploratory data analysis and differences between participant groups were tested on centered log-ratio–transformed values from the original data. The Selbal algorithm (27) was employed to determine the optimal combination of GPs for predicting death. Specifically, GPs entered the model as balances, which are normalized logarithmic ratios between two subsets of components from the original data. The variable selection algorithm initiated with a search to identify the two GPs whose balance was most strongly associated with the response variable (*i.e.*, death), then the algorithm employed a forward selection process. At each step, the GP that most improved the area under the curve (AUC) of the model was added. This process continued until no further enhancement in classification accuracy could be achieved. The selected balances were included in a multivariate Cox regression model, and their statistical significance was tested using a log-likelihood ratio test.

The AUCs from the receiver operating characteristic (ROC) analysis were used to assess the GPs’ predictive performance. The ROC curves and the AUC, with 95% confidence intervals (CIs), were calculated using the pROC R package (28). In addition, we estimated the time-dependent AUC to assess the predictive performance of the chosen balances over time. Statistical analyses were performed in R (29), using the packages’ compositions (https://CRAN.R-project.org/package=compositions) and mgcv (30).

## Results

### All-Cause Mortality

During the follow-up, 130 of the 1516 participants died. The death incidence rate was 8.6% (95% CI, 7.2–10.1) after a mean follow-up of 7.01 years. The participants who died were older, predominantly men, had a higher BMI, lower physical activity, a higher proportion of metabolic syndrome, exsmoking, and high or heavy alcohol consumption. Moreover, they showed higher values of glycemic (FPG and HbA1c) and inflammatory (CRP, TNF-alpha, and ESR) markers, as well as a lower GFR, lower levels of total cholesterol, HDL cholesterol, and low-density lipoprotein cholesterol, and higher levels of aspartate aminotransferase. They also had higher prevalence of hypertension, diabetes mellitus, ischemic heart disease, heart failure, peripheral artery disease, stroke, and cancer (Table 1).

**Table 1:** Baseline characteristics of AEGIS participants stratified by alive or dead status

Variable	AEGIS (n = 1516)	Alive (n = 1386)	Dead (n = 130)	*P*
Age (years)^a^	52 [39, 67]	50 [38, 64]	76 [66, 82]	<0.001
Sex (female)^b^	837 (55.2%)	783 (56.5%)	54 (41.5%)	0.001
BMI (kg/m^2^)^a^	27.8 [24.6, 31.4]	27.6 [24.4, 31.2]	29.8 [26.3, 34.1]	<0.001
Smoking status^b^				0.000
Non	825 (54.4%)	757 (54.7%)	67 (51.5%)	
Ex	395 (26.1%)	344 (24.8%)	51 (39.2%)	
Smoker	296 (19.5%)	284 (20.5%)	12 (9.2%)	
Alcohol consumption^b^				0.090
Abstemious	546 (36.0%)	495 (35.7%)	50 (38.5%)	
Light drinker	598 (39.5%)	558 (40.3%)	40 (30.8%)	
Moderate	241 (15.9%)	218 (15.7%)	23 (17.7%)	
Heavy	131 (8.6%)	114 (8.2%)	17 (13.1%)	
Physical activity^b^				0.002
Low	596 (39.3%)	528 (38.1%)	68 (52.3%)	
Medium	552 (36.4%)	508 (36.7%)	44 (33.8%)	
High	368 (24.2%)	349 (25.2%)	18 (13.8%)	
Metabolic syndrome^b^	314 (20.7%)	265 (19.1%)	49 (37.7%)	<0.001
Diabetes mellitus^b^	183 (12.1%)	137 (9.9%)	46 (35.4%)	<0.001
FPG (mg/dl)^a^	89.0 [82.0, 100.0]	88.0 [81.0, 98.0]	100.5 [88.0, 117.0]	<0.001
HbA1c (%)^a^	5.4 [5.2, 5.7]	5.4 [5.2, 5.7]	5.7 [5.4, 6.3]	<0.001
Cholesterol (mg/dl)^a^	195.0 [169.0, 220.0]	195.0 [170.0, 222.0]	187.0 [164.2, 213.0]	0.011
LDL cholesterol (mg/dl)^a^	113.0 [94.0, 134.0]	114.0 [94.0, 135.0]	106.0 [86.0, 124.8]	0.003
HDL cholesterol (mg/dl)^a^	57.0 [47.0, 69.0]	58.0 [48.0, 69.0]	53.0 [45.0, 63.8]	0.001
ESR (mm/h)^a^	9.0 [5.0, 17.0]	9.0 [5.0, 16.0]	15.0 [7.8, 28.0]	<0.001
CRP (mg/dl)^a^	0.14 [0.04, 0.39]	0.13 [0.04, 0.36]	0.27 [0.10, 0.66]	<0.001
TNF-alpha (pg/ml)^a^	7.4 [6.1, 9.0]	7.2 [6.0, 8.7]	8.3 [7.0, 9.5]	<0.001
AST (UI/L)^a^	23.0 [19.0, 27.0	22.0 [19.0, 27.0]	24.0 [21.0, 28.0]	0.001
GFR (mL/min)^a^	100.4 [86.9, 115.1]	101.9 [88.3, 116.5]	88.2 [70.3, 100.0]	<0.001
Arterial hypertension^b^	486 (32.1%)	396 (28.6%)	90 (69.2%)	<0.001
Heart failure^b^	26 (1.7%)	11 (0.8%)	15 (11.5%)	<0.001
IHD^b^	65 (4.3%)	42 (3.0%)	23 (17.7%)	<0.001
PAD^b^	27 (1.8%)	15 (1.1%)	12 (9.2%)	<0.001
Stroke^b^	35 (2.3%)	22 (1.6%)	13 (10.0%)	<0.001
Cancer^b^	71 (4.7%)	53 (3.8%)	18 (13.8%)	<0.001

AST, aspartate aminotransferase; IHD, ischemic heart disease; LDL, low-density lipoprotein; PAD, peripheral artery disease.

a Median and [interquartile range].

b Absolute frequency and (percentage).

The participants who died showed a greater abundance of GP1, 2, 3, 5, 6, 7, 10, 16, 41, and 46 and a lesser abundance of GP8, 9, 14, 17, 18, 20, 22, 24, 25, 26, 27, 29, 30, 31, 33, 34, 35, and 36 than those who were alive (Supplementary Table).

**Fig. 1:**
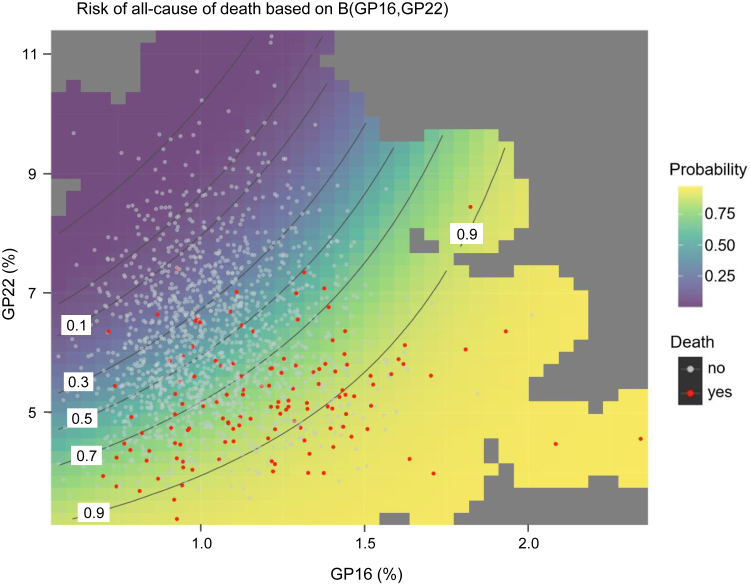
**Effect of GP16 and GP22 on the incidence of all-cause death using a balance (B[GP16,GP22]).** B, balance; GP, glycome peak.

**Fig. 2:**
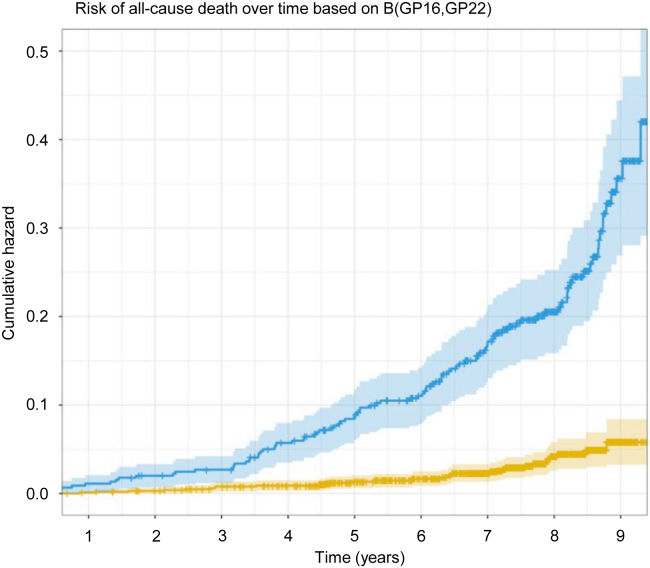
**Cumulative incidence of all-cause death stratified by (B[GP16,GP22]).** B, balance; GP, glycome peak.

**Fig. 3:**
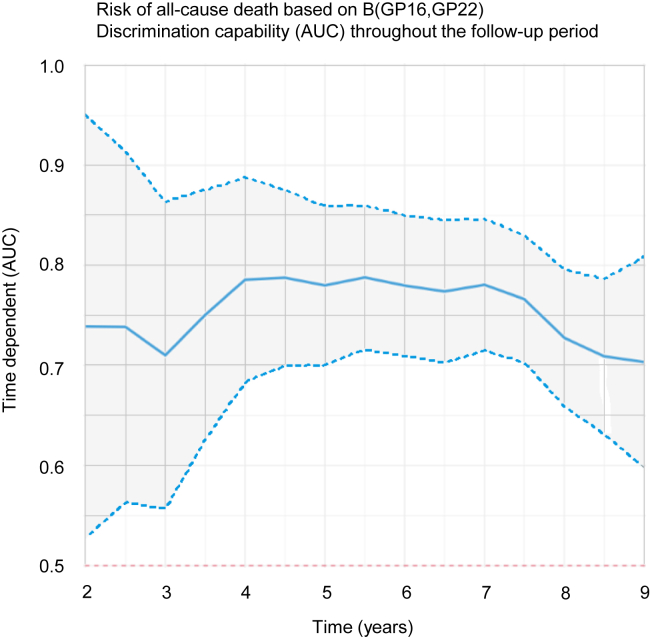
**Discrimination capability of GP16 and GP22 (B[GP16,GP22]) for the incidence of all-cause death throughout the follow-up period.** AUC, area under the curve; B, balance; GP, glycome peak.

The *selbal* algorithm identified the balance (B): B(GP16,GP22) (Supplementary Fig. S1) as the optimal balance for predicting all-cause mortality, with an AUC of 0.810 (95% CI, 0.773–0.847). Higher relative values of GP16 to GP22 showed a higher risk of death (Fig. 1). Those participants with a B(GP16,GP22) value higher than or equal to 1.15 displayed a 30.0% (95% CI, 23.6–35.8) cumulative incidence of death after 9 years, whereas those with lower values had a cumulative incidence of 5.6% (95% CI, 3.2–8.0) (Fig. 2). The predictive accuracy of GP16 and 22 remained stable, near 0.80, throughout the follow-up period (Fig. 3). *N*-glycome is therefore a potential predictor of all-cause death, both in the short and long term.

The effect of B(GP16,GP22) on mortality, adjusted for common risk factors, was estimated using multivariate Cox regression models and was independent of age, sex, and other factors, which were chosen using an elastic net variable selection algorithm (Table 2).

**Table 2:** Effect of risk factors on all-cause mortality estimated using a multivariate Cox regression

Predictor variables	B(GP16,GP22)	Age + sex HR	Age + sex + B(GP16,GP22)	Risk factors	Risk factors + B(GP16,GP22)
	HR (95% CI)	HR (95% CI)	HR (95% CI)	HR (95% CI)	HR (95% CI)
B(GP16,GP22)	1.62 (1.49–1.76)	—	1.31 (1.19–1.45)	—	1.30 (1.17–1.43)
Age (years)	—	1.10 (1.09–1.12)	1.09 (1.07–1.10)	1.10 (1.08–1.12)	1.09 (1.07–1.11)
Sex (ref: female)	—	2.28 (1.60–3.25)	2.51 (1.76–3.58)	1.64 (1.04–2.60)	1.86 (1.17–2.96)
BMI (kg/m^2^)	—	—	—	1.01 (0.97–1.05)	1.01 (0.97–1.05)
Exsmoker	—	—	—	1.57 (1.01–2.45)	1.52 (0.96–2.41)
Smoker	—	—	—	1.56 (0.74–3.25)	1.56 (0.75–3.25)
HDL cholesterol (mmol/L)	—	—	—	0.99 (0.97–1.00)	0.99 (0.97–1.00)
ESR (mm/h)	—	—	—	1.02 (1.01–1.03)	1.02 (1.00–1.03)
AST (UI/L)	—	—	—	1.03 (1.02–1.05)	1.03 (1.02–1.05)
Heart failure	—	—	—	3.06 (1.74–5.37)	4.07 (2.31–7.16)
Log-likelihood ratio test	—	<0.001		<0.001	

Risk factors of all-cause death were selected based on the elastic net regularization method.

AST, aspartate aminotransferase; HR, hazard ratio; ref, reference.

### Cancer Mortality

Deaths because of cancer were 43 (33%) out of 130. The cancer death incidence rate was 2.8% (95% CI, 2.1–3.8) after a mean follow-up of 7.01 years. The participants who died from cancer were older, had a higher BMI, and higher proportion of exsmoking and metabolic syndrome. Moreover, they showed higher values of glycemic (FPG and HbA1c) and inflammatory (CRP, TNF-alpha, and ESR) markers, as well as a lower GFR, lower levels of HDL cholesterol, and a higher prevalence of hypertension, diabetes mellitus, ischemic heart disease, peripheral artery disease, and cancer (Table 3).

**Table 3:** Baseline characteristics of AEGIS participants stratified by noncancer death or cancer death status

Variable	Noncancer death (n = 1472)	Cancer death (n = 43)	*p*
Age (years)^a^	52.0 [38.8, 66.0]	66.0 [60.0, 78.5]	<0.001
Sex (female)^b^	819 (55.6%)	18 (41.9%)	0.102
BMI (kg/m^2^)^a^	27.7 [24.5, 31.3]	30.0 [28.0, 34.6]	<0.001
Smoking status^b^			0.055
Non	805 (54.7%)	19 (44.2%)	
Ex	377 (25.6%)	18 (41.9%)	
Smoker	290 (19.7%)	6 (14.0%)	
Alcohol consumption^b^			0.998
Abstemious	530 (36.0%)	15 (34.9%)	
Light drinker	581 (39.5%)	17 (39.5%)	
Moderate	234 (15.9%)	7 (16.3%)	
Heavy	127 (8.6%)	4 (9.3%)	
Physical activity^b^			0.871
Low	578 (39.3%)	18 (41.9%)	
Medium	536 (36.4%)	16 (37.2%)	
High	358 (24.3%)	9 (20.9%)	
Metabolic syndrome^b^	295 (20.0%)	19 (44.2%)	<0.001
Diabetes mellitus^b^	170 (11.5%)	13 (30.2%)	<0.001
FPG (mg/dl)^a^	89.0 [82.0, 99.0]	98.0 [87.0, 113.5]	<0.001
HbA1c (%)^a^	5.4 [5.2, 5.7]	5.7 [5.4, 6.0]	<0.001
Cholesterol (mg/dl)^a^	195.0 [169.0, 220.0]	195.0 [173.0, 216.0]	0.612
LDL cholesterol (mg/dl)^a^	114.0 [94.0, 135.0]	107.0 [96.0, 123.5]	0.138
HDL cholesterol (mg/dl)^a^	58.0 [47.0, 69.0]	49.0 [40.5, 63.5]	0.013
ESR (mm/h)^a^	9.0 [5.0, 16.0]	16.0 [7.2, 25.5]	0.001
CRP (mg/dl)^a^	0.1 [0.0, 0.4]	0.3 [0.1, 0.8]	0.002
TNF-alpha (pg/ml)^a^	7.4 [6.1, 8.9]	8.9 [6.9, 10.7]	0.001
AST (UI/L)^a^	23.0 [19.0, 27.0]	23.0 [21.0, 28.0]	0.101
GFR (mL/min)^a^	100.6 [87.1, 115.1]	96.0 [78.7, 107.2]	0.047
Arterial hypertension^b^	461 (31.3%)	25 (58.1%)	<0.001
Heart failure^b^	25 (1.7%)	1 (2.3%)	1.000
IHD^b^	59 (4.0%)	6 (14.0%)	0.005
PAD^b^	22 (1.5%)	5 (11.6%)	<0.001
Stroke^b^	33 (2.2%)	2 (4.7%)	0.602
Cancer^b^	61 (4.1%)	10 (23.3%)	<0.001

AST, aspartate aminotransferase; IHD, ischemic heart disease; LDL, low-density lipoprotein; PAD, peripheral artery disease.

a Median and [interquartile range].

b Absolute frequency and (percentage).

The participants who died because of cancer showed a greater abundance of GP5, 6, 16, and 37 and lesser abundance of GP4, 14, 22, 24, 27, and 33 than those who did not die because of cancer (Supplementary Table).

**Fig. 4:**
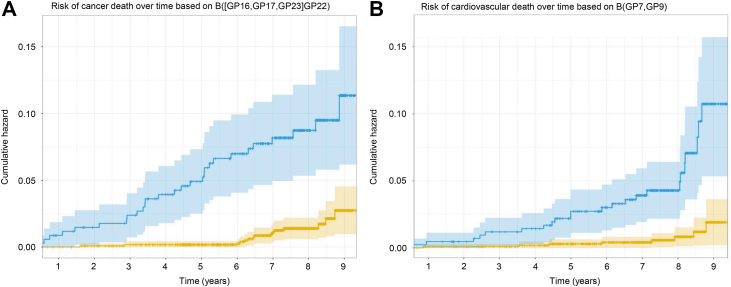
**Cumulative incidence of cancer death stratified by (B[GP16,GP17,GP23]GP22) (*A*); cumulative incidence of cardiovascular death stratified by B[GP7,GP9) (*B*).** B, balance; GP, glycome peak.

**Fig. 5:**
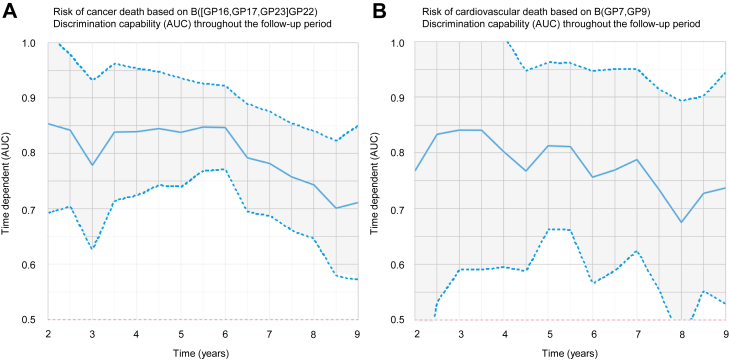
**Discrimination capability of GP16, GP17, GP23, and GP22 (B[GP16,GP17,GP22]GP23) for the incidence of cancer death throughout the follow-up period (*A*); discrimination capability of GP7 and GP9 (B[GP7,GP9]) for the incidence of cardiovascular death throughout the follow-up period (*B*).** AUC, area under the curve; B, balance; GP, glycome peak.

The *selbal* algorithm identified B([GP16,GP17,GP23],GP22) (Supplementary Figs. S1 and S2) as the optimal balance for predicting death because of cancer, with an AUC of 0.786 (95% CI, 0.728–0.843). Higher relative values of GP16, GP17, and GP23 to GP22 showed a higher risk of death by cancer. Those participants with B([GP16,GP17,GP23],GP22) ≥1.12 displayed a 10.7% (95% CI, 5.9–15.2) cumulative incidence of death by cancer after 9 years, whereas those with lower values had a cumulative incidence of 2.7% (95% CI, 0.9–4.4) (Fig. 4*A*). The predictive accuracy of GP16, 17, 23, and 22 decreased throughout the follow-up period (Fig. 5*A*). *N*-glycome is therefore a better potential predictor of cancer death in the short term.

The effect of B([GP16,GP17,GP23],GP22) on mortality, adjusted for common risk factors, was estimated using multivariate Cox regression models and was independent of age, sex, and other factors, which were chosen using an elastic net variable selection algorithm (Table 4).

**Table 4:** Effect of risk factors on cancer mortality estimated using a multivariate Cox regression

Predictor variables	B([GP16,GP17,GP23]GP22)	Age + sex	Age + sex + B([GP16,GP17,GP23]GP22)	Risk factors	Risk factors + B([GP16,GP17,GP23]GP22)
	HR (95% CI)	HR (95% CI)	HR (95% CI)	HR (95% CI)	HR (95% CI)
B([GP16,GP17,GP23]GP22)	1.64 (1.41–1.91)	—	1.50 (1.25–1.80)	—	1.44 (1.17–1.78)
Age (years)	—	1.06 (1.03–1.08)	1.03 (1.01–1.06)	1.03 (1.01–1.06)	1.02 (0.99–1.05)
Sex (ref: female)	—	2.10 (1.13–3.90)	2.33 (1.25–4.34)	1.67 (0.78–3.59)	1.63 (0.75–3.53)
BMI (kg/m^2^)	—	—	—	1.04 (0.98–1.11)	1.03 (0.96–1.09)
ESR (mm/h)	—	—	—	1.02 (1.00–1.04)	1.01 (0.99–1.03)
Arterial hypertension	—	—	—	1.03 (1.01–1.05)	1.03 (1.01–1.05)
Cancer	—	—	—	3.71 (1.74–7.90)	4.04 (1.91–8.51)
Log-likelihood ratio test	—	<0.001		<0.001	

Risk factors of cancer death were selected based on the elastic net regularization method.

HR, hazard ratio; ref, reference.

### Cardiovascular Mortality

Deaths because of a cardiovascular cause were 32 (25%) out of 130. The cardiovascular death incidence rate was 2.1% (95% CI, 1.4–3.0) after a mean follow-up of 7.01 years. The participants who died of a cardiovascular cause were older, predominantly men, had a higher BMI, lower physical activity, a higher proportion of metabolic syndrome, and high or heavy alcohol consumption. Moreover, they showed higher values of glycemic (FPG and HbA1c) and inflammatory (CRP, TNF-alpha, and ESR) markers, as well as a lower GFR, and higher prevalence of hypertension, diabetes mellitus, ischemic heart disease, heart failure, peripheral artery disease, and stroke (Table 5).

**Table 5:** Baseline characteristics of AEGIS participants stratified by noncardiovascular death or cardiovascular death status

Variable	Noncardiovascular death (n = 1484)	Cardiovascular death (n = 32)	*p*
Age (years)^a^	52.0 [39.0, 66.0]	76.5 [69.8, 82.0]	<0.001
Sex (female)^b^	826 (55.7%)	11 (34.4%)	0.026
BMI (kg/m^2^)^a^	27.7 [24.6, 31.3]	29.9 [27.3, 33.1]	0.006
Smoking status^b^			0.271
Non	806 (54.3%)	18 (56.2%)	
Ex	384 (25.9%)	11 (34.4%)	
Smoker	293 (19.8%)	3 (9.4%)	
Alcohol consumption^b^			0.040
Abstemious	539 (36.3%)	6 (18.8%)	
Light drinker	586 (39.5%)	12 (37.5%)	
Moderate	233 (15.7%)	8 (25.0%)	
Heavy	125 (8.4%)	6 (18.8%)	
Physical activity^b^			0.005
Low	575 (38.8%)	21 (65.6%)	
Medium	543 (36.6%)	9 (28.1%)	
High	365 (24.6%)	2 (6.2%)	
Metabolic syndrome^b^	298 (20.1%)	16 (50.0%)	<0.001
Diabetes mellitus^b^	167 (11.3%)	16 (50.0%)	<0.001
FPG (mg/dl)^a^	88.0 [82.0, 99.0]	109.5 [97.5, 136.5]	<0.001
HbA1c (%)^a^	5.4 [5.2, 5.7]	6.2 [5.8, 7.3]	<0.001
Cholesterol (mg/dl)^a^	195.0 [169.0, 221.0]	179.0 [165.2, 212.0]	0.151
LDL cholesterol (mg/dl)^a^	114.0 [94.0, 134.8]	105.5 [85.0, 131.2]	0.228
HDL cholesterol (mg/dl)^a^	58.0 [47.0, 69.0]	54.5 [45.0, 63.2]	0.197
ESR (mm/h)^a^	9.0 [5.0, 16.0]	19.0 [9.0, 30.0]	<0.001
CRP (mg/dl)^a^	0.1 [0.0, 0.4]	0.3 [0.1, 0.9]	0.002
TNF-alpha (pg/ml)^a^	7.4 [6.0, 8.9]	9.9 [7.7, 11.0]	0.001
AST (UI/L)^a^	23.0 [19.0, 27.0]	23.5 [20.0, 33.2]	0.199
GFR (mL/min)^a^	101.1 [87.1, 115.2]	88.2 [73.2, 93.8]	<0.001
Arterial hypertension^b^	462 (31.2%)	24 (75.0%)	<0.001
Heart failure^b^	20 (1.3%)	6 (18.8%)	<0.001
IHD^b^	58 (3.9%)	7 (21.9%)	<0.001
PAD^b^	22 (1.5%)	5 (15.6%)	<0.001
Stroke^b^	31 (2.1%)	4 (12.5%)	0.001
Cancer^b^	66 (4.5%)	5 (15.6%)	0.011

AST, aspartate aminotransferase; IHD, ischemic heart disease; LDL, low-density lipoprotein; PAD, peripheral artery disease.

a Median and [interquartile range].

b Absolute frequency and (percentage).

The participants who died because of a cardiovascular cause showed a greater abundance of GP2, 3, 5, 6, 7, and 16 and lesser abundance of GP14, 22, 25, and 27 than those who did not die because of a cardiovascular cause (Supplementary Table).

The *selbal* algorithm identified B(GP7,GP9) (Supplementary Fig. S3) as the optimal balance for predicting cardiovascular death, with an AUC of 0.747 (95% CI, 0.645–0.850). Higher relative values of GP7 to GP9 showed a higher risk of cardiovascular death. Those participants with B(GP7,GP9) ≥1.53 displayed a 10.2% (95% CI, 5.2–14.9) cumulative incidence of cardiovascular death after 9 years, whereas those with lower values had a cumulative incidence of 1.89% (95% CI, 1.9–3.5) (Fig. 4*B*). The time-dependent AUC for B(GP7,GP9) showed high variability with broad confidence bands; however, it tended to decrease over time (Fig. 5*B*).

The effect of B(GP7,GP9) on mortality, adjusted for common risk factors, was estimated using multivariate Cox regression models and was independent of age, sex, and other factors, which were chosen using an elastic net variable selection algorithm (Table 6).

**Table 6:** Effect of risk factors on cardiovascular mortality estimated using a multivariate Cox regression

Predictor variables	B(GP7,GP9)	Age + sex	Age + sex + B(GP7,GP9)	Risk factors	Risk factors + B(GP7,GP9)
	HR (95% CI)	HR (95% CI)	HR (95% CI)	HR (95% CI)	HR (95% CI)
B(GP7,GP9)	1.33 (1.21–1.46)	—	1.16 (1.06–1.27)	—	1.22 (1.10–1.36)
Age (years)	—	1.14 (1.10–1.19)	1.12 (1.08–1.16)	1.13 (1.08–1.18)	1.11 (1.06–1.16)
Sex (ref: female)	—	3.36 (1.59–7.09)	2.87 (1.35–6.12)	2.71 (1.13–6.51)	2.55 (1.06–6.10)
BMI (kg/m^2^)	—	—	—	1.04 (0.96–1.13)	1.04 (0.96–1.13)
HAC (>280g/week)	—	—	—	4.14 (1.14–15.09)	3.86 (1.06–14.10)
ESR (mm/h)	—	—	—	1.03 (1.01–1.06)	1.02 (1.00–1.05)
AST (UI/L)	—	—	—	1.03 (1.00–1.06)	1.04 (1.00–1.07)
Heart failure	—	—	—	5.85 (2.27–15.07)	8.82 (3.29–23.64)
Stroke		—	—	3.24 (1.04–10.08)	3.95 (1.28–12.25)
Log-likelihood ratio test	—	<0.001		<0.001	

Risk factors of cardiovascular death were selected based on the elastic net regularization method.

AST, aspartate aminotransferase; HAC, heavy alcohol consumption; HR, hazard ratio; ref, reference.

## Discussion

The present study showed that the interaction between two TSNG GPs displayed a strong association with all-cause death risk. High levels of GP16 predisposed participants to death, whereas high levels of GP22 were protective against death. Furthermore, the balance between GP16 and GP22 improved the prediction of classical risk factors for all-cause mortality.

GP16 is composed of seven different glycans (Supplementary Fig. S1), three of them relatively abundant (22): A2[3]BG1S[3]1 (constituting approximately 31.5% of the total GP16) and A2[3]BG1S[6]1 (24.1%) (both sharing the same biantennary, bisected, monogalactosylated, and monosialylated structure, with the difference of the sialic acid linkage type); and M7 D1 (29.6%), an oligomannose oligosaccharide composed of two *N*-acetylglucosamines with seven mannose residues. The next most abundant glycans of GP16 are FA2[6]G1S[3]1 (8.3%) and FA2[6]G1S[6]1 (6.5%), which also share the same structure, except for sialic acid linkage type*.* The two remaining glycans are M4A1G1S[3]1 (<1%) and M4A1G1S[6]1 (<1%), hybrid oligosaccharides that only contain mannose residues on one branch of the core (4 mannose in the whole structure). GP22 is composed of three different glycans (Supplementary Fig. S1): FA2G2S[6]1 (constituting approximately 54.3% of the total GP16), FA2G2S[3]1 (37.2%), and M8 D1, D3 (8.4%) (22).

The predictive ability of GP16 and GP22 could depend on just one glycan in each cluster that is attached to a particular protein (22), or it could have a more complex mechanism. The highest risk for all-cause mortality was found in those participants with high GP16 levels and low GP22 levels, and the lowest risk was in those who had low GP16 levels and high GP22 levels (Fig. 1). Therefore, GPs 16 and 22 might act in a dependent and complementary way in the population sample for mechanisms related to all-cause mortality.

The role played by the glycans that comprise GP16 and GP22 is unknown. They are probably bound to many proteins, including immunoglobulins A, D, E, G, M, and acute-phase proteins (31, 32), for example, alpha-2-macrogobulin (32), a macromolecule that inhibits proteases, achieving protection against structural damage during inflammation, among other functions (33). However, their association with mortality has not been explored, and the mechanisms involved remain enigmatic. In a previous study we conducted in the same population, we found that individuals who showed a predominance of simple *N*-glycans (including GP16) were older, had higher concentrations of glycation markers and some inflammatory markers, a lower GFR, and greater comorbidity than individuals who showed a predominance of more complex *N*-glycans (including GP22) (34). To the best of our knowledge, no study has investigated all-cause death. Only a small number have examined the association between total serum/plasma *N*-glycome and cancer or cardiovascular diseases; few have analyzed its association with prognosis, as we will discuss below.

We have found that in addition to GP16, high levels of GP17 and GP23 predisposed participants to death from cancer, whereas high levels of GP22 were protective against it. GP17 is composed of four different glycans (Supplementary Fig. S2): FA2[3]G1S[6]1 (constituting approximately 43.8% of the total GP17), FA2[3]G1S[3]1 (35.6%), FA2[6]BG1S[6]1 (15.1%), and FA2[6]BG1S[3]1 (5.5%). GP23 is composed of FA2BG2S[3]1 (51.8%) and FA2BG2S[6]1 (48.2%) (22). *N*-glycans in GP17 and GP23 can also bind to immunoglobulins (31, 32). GP23 abundance was associated with levels of immunoglobulin M and CD5L (35), a protein whose overexpression in tumor-associated macrophages correlated with poorer patient prognosis, and it could be an immune checkpoint in macrophages (36).

Some previous studies have shown some data in line with our results. The analysis of TSNG in patients with breast cancer had demonstrated that GP22 was significantly associated with specific survival (37). A decrease of GP22 was a constant biomarker across all stages of colorectal cancer (38); whereas, GP23 showed higher abundance associated with poor prognosis in patients with breast cancer (39). *N*-glycans corresponding to our GP16 (GP18 in the original article) were higher in patients with significant prostate cancer than in patients with indolent cancer (40). M4A1G1S1 (part of our GP16) was increased in patients with colorectal cancer, whereas FA2G2S1 (part of our GP22) was decreased (41). However, other studies showed different data, probably because of cancer encompassing a large group of diseases, where multiple pathophysiological mechanisms are involved. In fact, serum *N*-glycan profiles can differ in cancer subtypes of the same organ (42).

In addition, high levels of GP7 correlated with death from cardiovascular disease, whereas high levels of GP9 correlated with protection against it. Both GP7 and GP9 are composed of single glycans (Supplementary Fig. S3): A2[6]BG1 and FA2[3]G1, respectively (22). Both GP7 and GP9 can bind immunoglobulins and can bind acute-phase proteins (31, 32). A2[6]BG1 abundance correlated with levels of low-affinity immunoglobulin gamma Fc region receptor III-B (35), which might act as a damper of Fc-dependent immune reactions (43).

An interesting previous study had analyzed plasma *N*-glycans as biomarkers of cardiometabolic risk (9). It showed FA2[3]G1 (our GP9) was inversely associated with incident cardiovascular events in women; whereas *N*-glycans corresponding to our GP22 (GP16 in the original article) were associated with lower risk of cardiovascular events in men. However, immunoglobulin G, the most abundant *N*-glycosylated protein in serum, has been more studied in relation to cardiovascular risk than TSNG. In this regard, FA2G2S1 (*N*-glycan in our GP22) was negatively associated with both atherosclerotic cardiovascular risk (44), and FA2[3]G1 (our GP9) was negatively associated with both atherosclerotic cardiovascular risk (44) and cardiovascular disease incidence in women (45). In another study, FA2[3]G1 (our GP9) was negatively associated with ischemic stroke in men (46).

The effect of corresponding balances of glycans on all-cause death, cancer death, and cardiovascular death was independent of age, sex, and other risk factors, which were chosen using an elastic net variable selection algorithm. Furthermore, the effect of the balances was greater than the effect of most risk factors, including age (Tables 2, 4 and 6). The balance between GP16 and GP22 displayed a diagnostic accuracy near 0.80 throughout the follow-up period (Fig. 3). However, B([GP16,GP17,GP23]GP22) displayed a diagnostic accuracy that decreased throughout the follow-up period (Fig. 5*A*), and the diagnostic accuracy of B(GP7,GP9) also tended to decrease throughout the follow-up period (Fig. 5*B*). Therefore, recalculated balances focused on predicting both cancer and cardiovascular death risk for a shorter time might improve diagnostic accuracy; however, more data are needed.

These glycans might reflect an epiphenomenon or perhaps the glycosylation signature of key proteins involved in pathophysiological mechanisms that increase lethality, either directly or indirectly. In any case, the results we show make further research interesting and mandatory, bearing in mind that death is the most significant primary endpoint in clinical research and population-based studies.

The main strengths of the study are its population-based design, including a wide age range of participants and random sampling; use of a reliable method for the determination of TSNG, and the wide availability of data, with full access to medical records for follow-up. The fundamental limitations of the study are its small sample size and the need to validate the predictive ability of TSNG in other populations, including ethnicities other than White.

To the best of our knowledge, this is the first study investigating TSNG to predict mortality in a general adult population. We identified GPs that prediposed individuals to death and GPs that protected against death. The balances between these GPs predicted all-cause mortality, cancer mortality, and cardiovascular mortality over time. Their predictive powers had an independent and additive effect on classical prediction factors and contributed significantly to improving prognostic tools. Future studies, both in the general population and in patients, could elucidate the role of these *N*-glycans in mortality and their usefulness in the management of health and disease.

## Data Availability

The datasets generated and/or analyzed during the current study are not publicly available because of Spanish law restrictions but are available from the corresponding author on reasonable request.

## Supplemental Data

This article contains supplemental data.

## Conflict of Interest

Iago Carballo, Óscar Lado-Baleato, Francisco Gude, and Arturo González-Quintela have applied for a patent related to the results described in this work: a method for predicting the risk of mortality. All other authors declare no competing interests.

## References

[1] Barchi J.J. (2021).

[2] Moremen K.W., Tiemeyer M., Nairn A.V. (2012). Vertebrate protein glycosylation: diversity, synthesis and function. Nat. Rev. Mol. Cell Biol..

[3] de Vroome S.W., Holst S., Girondo M.R., van der Burgt Y.E.M., Mesker W.E., Tollenaar R.A.E.M. (2018). Serum *N*-glycome alterations in colorectal cancer associate with survival. Oncotarget.

[4] Saldova R., Royle L., Radcliffe C.M., Abd Hamid U.M., Evans R., Arnold J.N. (2007). Ovarian cancer is associated with changes in glycosylation in both acute-phase proteins and IgG. Glycobiology.

[5] Vreeker G.C.M., Hanna-Sawires R.G., Mohammed Y., Bladergroen M.R., Nicolardi S., Dotz V. (2020). Serum *N*-Glycome analysis reveals pancreatic cancer disease signatures. Cancer Med..

[6] Sato Y., Nakata K., Kato Y., Shima M., Ishii N., Koji T. (1993). Early recognition of hepatocellular carcinoma based on altered profiles of alpha-fetoprotein. N. Engl. J. Med..

[7] Memarian E., t Hart L.M., Slieker R.C., Lemmers R.F.L., van der Heijden A.A., Rutters F. (2021). Plasma protein *N*-glycosylation is associated with cardiovascular disease, nephropathy, and retinopathy in type 2 diabetes. BMJ Open Diabetes Res. Care.

[8] Testa R., Vanhooren V., Bonfigli A.R., Boemi M., Olivieri F., Ceriello A. (2015). *N*-glycomic changes in serum proteins in type 2 diabetes mellitus correlate with complications and with metabolic syndrome parameters. PLoS One.

[9] Wittenbecher C., Štambuk T., Kuxhaus O., Rudman N., Vučković F., Štambuk J. (2020). Plasma *N*-glycans as emerging biomarkers of cardiometabolic risk: a prospective investigation in the EPIC-potsdam cohort study. Diabetes Care.

[10] (2024). WHO Mortality Database.

[11] Sung H., Ferlay J., Siegel R.L., Laversanne M., Soerjomataram I., Jemal A. (2021). Global cancer statistics 2020: GLOBOCAN estimates of incidence and mortality worldwide for 36 cancers in 185 countries. CA Cancer J. Clin..

[12] Ebell M.H., Thai T.N., Royalty K.J. (2018). Cancer screening recommendations: an international comparison of high income countries. Public Health Rev..

[13] Polonsky T.S., Greenland P. (2012). CVD screening in low-risk, asymptomatic adults: clinical trials needed. Nat. Rev. Cardiol..

[14] Bunker J.P. (1990). Institute of Medicine (US) Committee on Technological Innovation in Medicine. Modern Methods of Clinical Investigation: Medical Innovation at the Crossroads: Volume I.

[15] Expert panel on detection, evaluation and treatment of high blood cholesterol in adults (2001). Executive summary of third report of the National Cholesterol Education Program (NCEP) expert panel on detection, evaluation, and treatment of high blood cholesterol in adults (Adult Treatment Panel III). JAMA.

[16] Gual A., Martos A.R., Lligoña A., Llopis J.J. (1999). Does the concept of a standard drink apply to viticultural societies?. Alcohol Alcohol..

[17] Román-Viñas B., Lourdes Ribas-Barba L., Ngo J. (2013). Validity of the international physical activity questionnaire in the Catalan population (Spain). Gac Sanit.

[18] Alende-Castro V., Alonso-Sampedro M., Vazquez-Temprano N., Tuñez C., Rey D., García-Iglesias C. (2019). Factors influencing erythrocyte sedimentation rate in adults: new evidence for an old test. Medicine.

[19] Levey A.S., Coresh J., Greene T., Stevens L.A., Zhang Y.L., Hendriksen S. (2006). Using standardized serum creatinine values in the modification of diet in renal disease study equation for estimating glomerular filtration rate. Ann. Intern. Med..

[20] O'Flaherty R., Simon Á., Alonso-Sampedro M., Sánchez-Batán S., Fernández-Merino C., Gude F. (2022). Changes in serum *N*-glycome for risk drinkers: a comparison with standard markers for alcohol abuse in men and women. Biomolecules.

[21] Stöckmann H., O’Flaherty R., Adamczyk B., Saldova R., Rudd P.M. (2015). Automated, high-throughput serum glycoprofiling platform. Integr. Biol. (Camb).

[22] Saldova R., Asadi Shehni A., Haakensen V.D., Steinfeld I., Hilliard M., Kifer I. (2014). Association of *N*-glycosylation with breast carcinoma and systemic features using high-resolution quantitative UPLC. J. Proteome Res..

[23] Cheng K., Zhou Y., Neelamegham S. (2016). DrawGlycan-SNFG: a robust tool to render glycans and glycopeptides with fragmentation information. Glycobiology.

[24] Neelamegham S., Aoki-Kinoshita K., Bolton E., Frank M., Lisacek F., Lütteke T. (2019). Updates to the symbol nomenclature for glycans guidelines. Glycobiology.

[25] Zhao S., Walsh I., Abrahams J., Royle L., Nguyen-Khuong T., Spencer D. (2018). GlycoStore: a database of retention properties for glycan analysis. Bioinformatics.

[26] Royle L., Campbell M.P., Radcliffe C.M., White D.M., Harvey D.J., Abrahams J.L. (2008). HPLC-based analysis of serum N-glycans on a 96-well plate platform with dedicated database software. Anal. Biochem..

[27] Rivera-Pinto J., Egozcue J.J., Pawlowsky-Glahn V., Paredes R., Noguera-Julian M., Calle M.L. (2018). Balances: a new perspective for microbiome analysis. MSystems.

[28] Robin X., Turck N., Hainard A., Tiberti N., Lisacek F., Sanchez J.C. (2011). pROC: an open-source package for R and S+ to analyze and compare ROC curves. BMC Bioinform..

[29] R Core Team (2023).

[30] Wood S.N. (2017).

[31] O'Flaherty R., Muniyappa M., Walsh I., Stöckmann H., Hilliard M., Hutson R. (2019). A robust and versatile automated glycoanalytical technology for serum antibodies and acute phase proteins: ovarian cancer case study. Mol. Cell Proteomics.

[32] Clerc F., Reiding K.R., Jansen B.C., Kammeijer G.S.M., Bondt A., Wuhrer M. (2016). Human plasma protein *N*-glycosylation. Glycoconj J..

[33] Vandooren J., Itoh Y. (2021). Alpha-2-macroglobulin in inflammation, immunity and infections. Front. Immunol..

[34] Lado-Baleato Ó., Torre J., O'Flaherty R., Alonso-Sampedro M., Carballo I., Fernández-Merino C. (2023). Age-related changes in serum *N*-glycome in men and women-clusters associated with comorbidity. Biomolecules.

[35] Suhre K., Trbojević-Akmačić I., Ugrina I., Mook-Kanamori D.O., Spector T., Graumann J. (2019). Fine-mapping of the human blood plasma *N*-glycome onto its proteome. Metabolites.

[36] Sanchez-Moral L., Paul T., Martori C., Font-Díaz J., Sanjurjo L., Aran G. (2023). Macrophage CD5L is a target for cancer immunotherapy. EBioMedicine.

[37] Haakensen V.D., Steinfeld I., Saldova R., Shehni A.A., Kifer I., Naume B. (2016). Serum *N*-glycan analysis in breast cancer patients--Relation to tumour biology and clinical outcome. Mol. Oncol..

[38] Doherty M., Theodoratou E., Walsh I., Adamczyk B., Stöckmann H., Agakov F. (2018). Plasma *N*-glycans in colorectal cancer risk. Sci. Rep..

[39] Terkelsen T., Haakensen V.D., Saldova R., Gromov P., Hansen M.K., Stöckmann H. (2018). *N*-glycan signatures identified in tumor interstitial fluid and serum of breast cancer patients: association with tumor biology and clinical outcome. Mol. Oncol..

[40] Gilgunn S., Murphy K., Stöckmann H., Conroy P.J., Murphy T.B., Watson R.W. (2020). Glycosylation in indolent, significant and aggressive prostate cancer by automated high-throughput *N*-glycan profiling. Int. J. Mol. Sci..

[41] Takei D., Harada K., Nouso K., Miyahara K., Dohi C., Matsushita H. (2022). Clinical utility of a serum glycome analysis in patients with colorectal cancer. J. Gastroenterol. Hepatol..

[42] Vreeker G.C.M., Vangangelt K.M.H., Bladergroen M.R., Nicolardi S., Mesker W.E., Wuhrer M. (2021). Serum *N*-glycan profiles differ for various breast cancer subtypes. Glycoconj J..

[43] Wang Y., Jönsson F. (2019). Expression, role, and regulation of neutrophil Fcγ receptors. Front. Immunol..

[44] Menni C., Gudelj I., Macdonald-Dunlop E., Mangino M., Zierer J., Bešić E. (2018). Glycosylation profile of immunoglobulin G is cross-sectionally associated with cardiovascular disease risk score and subclinical atherosclerosis in two independent cohorts. Circ. Res..

[45] Birukov A., Plavša B., Eichelmann F., Kuxhaus O., Hoshi R.A., Rudman N. (2022). Immunoglobulin G *N*-glycosylation signatures in incident type 2 diabetes and cardiovascular disease. Diabetes Care.

[46] Wang B.Y., Song M.S., Zhang J., Meng X.N., Xing W.J., Wang Y.X. (2023). A nested case-control study to explore the association between immunoglobulin G N-glycans and ischemic stroke. Biomed. Environ. Sci..

