# Supplementary material for: Serum N-Glycans as Independent Predictors of Death: A Prospective Investigation in the AEGIS Cohort
Source: Mol Cell Proteomics. 2025 Oct 15;24(12):101217. doi: 10.1016/j.mcpro.2025.101217 (PMC12701958; doi:10.1016/j.mcpro.2025.101217)
Supplement: Supplementary Figures [file mmc1.pdf]

Supplementary Fig. S1. Glycans that compound glycome peak 16 and glycome peak 22 (20,22,26)

GP	Glycan	Structure
GP16	A2[3]BG1S[3]1 (31.5%)[a]	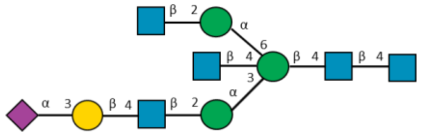
	A2[3]BG1S[6]1 (24.1%)[a]	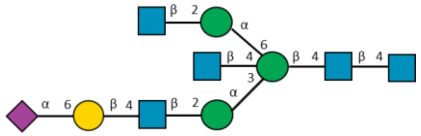
	M7 D1 (29.6%)[a]	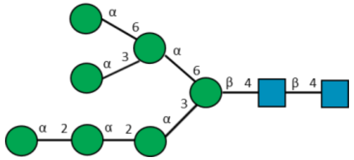
	FA2[6]G1S[3]1 (8.3%)[a]	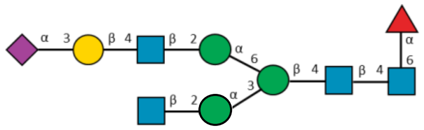
	FA2[6]G1S[6]1 (6.5%)[a]	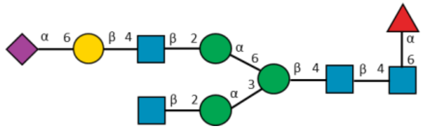
	M4A1G1S[3]1 (<1%)[a]	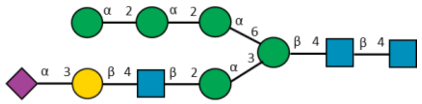
	M4A1G1S[6]1 (<1%)[a]	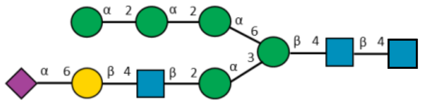

GP	Glycan	Structure
GP22	FA2G2S[6]1 (54.3%)[a]	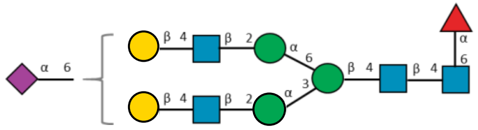
	FA2G2S[3]1 (37.2%)[a]	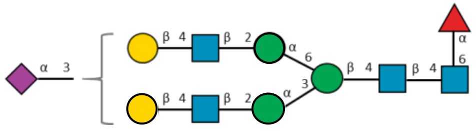
	M8 D1, D3 (8.4%)[a]	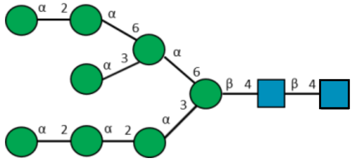

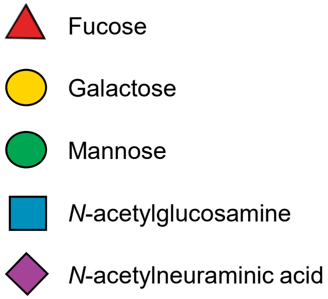

GP, glycome peak.

[a]Percentage of glycan structure in the GP.

Supplementary Fig. S2. Glycans that compound glycome peak 17 and glycome peak 23 (20,22,26)

GP17	FA2[3]G1S[6]1 (43.8%)[a]	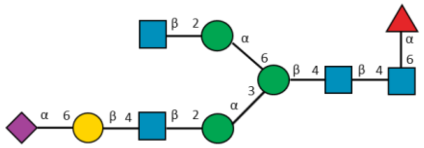
	FA2[3]G1S[3]1 (35.6%)[a]	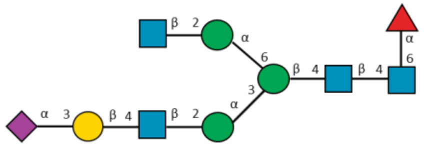
	FA2[6]BG1S[6]1 (15.1%)[a]	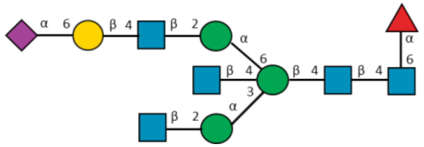
	FA2[6]BG1S[3]1 (5.5%)[a]	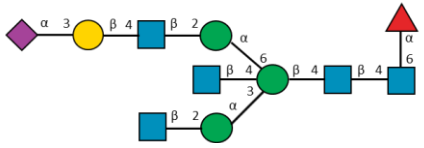

GP23	FA2BG2S[3]1 (51.8%)[a]	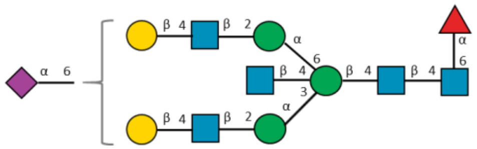
	FA2BG2S[6]1 (48.2%)[a]	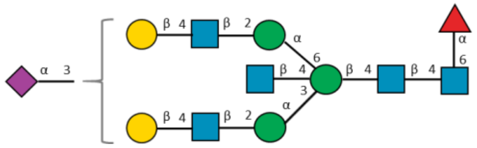

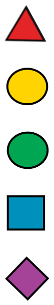

GP, glycome peak.

[a]Percentage of glycan structure in the GP.

Supplementary Fig. S3. Glycans that compound glycome peak 7 and glycome peak 9 (20,22,26)

GP7	A2[6]BG1	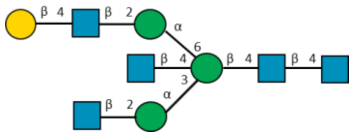
GP9	FA2[3]G1	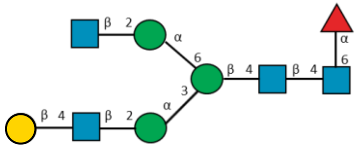

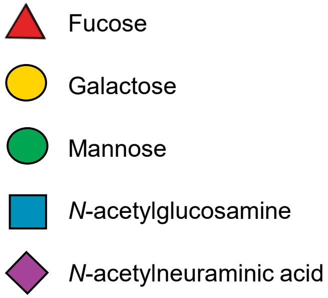

GP, glycome peak.

REFERENCES

20. R. O'Flaherty, Á. Simon, M. Alonso-Sampedro, S. Sánchez-Batán, C. Fernández-Merino, F. Gude, et al., Changes in serum *N*-glycome for risk drinkers: a comparison with standard markers for alcohol abuse in men and women, *Biomolecules*, **12**, 2022, 241.
22. R. Saldova, A. Asadi Shehni, V.D. Haakensen, I. Steinfeld, M. Hilliard, I. Kifer, et al., Association of *N*-glycosylation with breast carcinoma and systemic features using high-resolution quantitative UPLC, *J. Proteome Res*., **13**, 2014, 2314–2327.
26. L. Royle, M.P. Campbell, C.M. Radcliffe, D.M. White, D.J. Harvey, J.L. Abrahams, et al., HPLC-based analysis of serum N-glycans on a 96-well plate platform with dedicated database software, *Anal. Biochem*., **376**, 2008, 1–12.
