# Supplementary material for: Serum N-Glycans as Independent Predictors of Death: A Prospective Investigation in the AEGIS Cohort
Source: Mol Cell Proteomics. 2025 Oct 15;24(12):101217. doi: 10.1016/j.mcpro.2025.101217 (PMC12701958; doi:10.1016/j.mcpro.2025.101217)
Supplement: Supplementary Table [file mmc2.pdf]

Supplementary Table. Serum *N*-glycome peak abundance in alive versus all-cause death, noncancer death versus cancer death, and noncardiovascular death versus cardiovascular death

GP (%)	Alive (n=1386) (reference group)	All-cause death (n=130)	Noncancer death (n=1473) (reference group)	Cancer death (n=43)	Noncardiov. death (n=1484) (reference group)	Cardiov. death (n=32)
GP1	0.11 [0.08, 0.16]	0.15 [0.10, 0.21]	0.11 [0.08, 0.17]	0.13 [0.10, 0.18]	0.12 [0.08, 0.16]	0.15 [0.09, 0.23]
GP2	0.04 [0.03, 0.06]	0.05 [0.03, 0.08]	0.04 [0.03, 0.06]	0.04 [0.03, 0.07]	0.04 [0.03, 0.06]	0.06 [0.04, 0.08]
GP3	0.08 [0.05, 0.12]	0.12 [0.07, 0.20]	0.08 [0.05, 0.13]	0.08 [0.06, 0.15]	0.08 [0.05, 0.13]	0.13 [0.09, 0.20]
GP4	0.07 [0.05, 0.10]	0.07 [0.05, 0.10]	0.07 [0.05, 0.10]	0.06 [0.03, 0.08]	0.07 [0.05, 0.10]	0.07 [0.05, 0.12]
GP5	2.25 [1.61, 3.25]	3.21 [2.27, 4.76]	2.28 [1.62, 3.33]	2.77 [2.25, 4.11]	2.29 [1.63, 3.32]	3.63 [1.91, 4.54]
GP6	1.01 [0.81, 1.36]	1.34 [1.05, 1.83]	1.02 [0.82, 1.40]	1.14 [0.97, 1.60]	1.03 [0.82, 1.40]	1.43 [0.98, 1.93]
GP7	0.09 [0.07, 0.13]	0.12 [0.08, 0.17]	0.10 [0.07, 0.13]	0.10 [0.07, 0.15]	0.10 [0.07, 0.13]	0.13 [0.09, 0.20]
GP8	1.95 [1.54, 2.51]	1.91 [1.44, 2.51]	1.92 [1.53, 2.52]	1.85 [1.45, 2.36]	1.91 [1.53, 2.51]	2.04 [1.34, 2.47]
GP9	1.05 [0.82, 1.37]	1.04 [0.74, 1.32]	1.05 [0.82, 1.37]	0.98 [0.77, 1.29]	1.05 [0.82, 1.37]	1.04 [0.60, 1.24]
GP10	0.66 [0.53, 0.85]	0.82 [0.59, 1.00]	0.67 [0.54, 0.87]	0.73 [0.58, 0.94]	0.67 [0.54, 0.87]	0.83 [0.59, 0.99]
GP11	0.58 [0.48, 0.70]	0.63 [0.52, 0.84]	0.58 [0.48, 0.71]	0.63 [0.52, 0.78]	0.58 [0.48, 0.71]	0.61 [0.49, 0.86]
GP12	0.32 [0.24, 0.41]	0.35 [0.27, 0.43]	0.32 [0.24, 0.41]	0.31 [0.24, 0.39]	0.32 [0.24, 0.41]	0.34 [0.29, 0.43]
GP13	0.08 [0.06, 0.10]	0.09 [0.07, 0.11]	0.08 [0.06, 0.10]	0.09 [0.07, 0.10]	0.08 [0.06, 0.10]	0.08 [0.05, 0.11]
GP14	2.78 [2.27, 3.46]	2.39 [1.85, 2.90]	2.76 [2.25, 3.44]	2.50 [1.97, 2.87]	2.76 [2.25, 3.43]	2.25 [1.83, 2.73]
GP15	0.50 [0.40, 0.63]	0.50 [0.40, 0.65]	0.50 [0.40, 0.63]	0.50 [0.40, 0.61]	0.50 [0.40, 0.63]	0.52 [0.44, 0.65]
GP16	1.01 [0.89, 1.17]	1.21 [0.97, 1.39]	1.02 [0.90, 1.18]	1.12 [0.99, 1.34]	1.02 [0.90, 1.18]	1.22 [0.99, 1.39]
GP17	1.06 [0.87, 1.22]	1.05 [0.90, 1.19]	1.05 [0.88, 1.21]	1.09 [0.91, 1.20]	1.06 [0.88, 1.21]	1.01 [0.90, 1.20]
GP18	0.18 [0.14, 0.22]	0.17 [0.14, 0.21]	0.18 [0.13, 0.22]	0.17 [0.14, 0.21]	0.18 [0.14, 0.22]	0.19 [0.14, 0.21]
GP19	7.51 [6.90, 8.14]	7.58 [7.08, 8.28]	7.52 [6.90, 8.15]	7.57 [7.17, 8.21]	7.53 [6.91, 8.16]	7.45 [7.03, 7.99]
GP20	0.65 [0.58, 0.71]	0.64 [0.56, 0.73]	0.65 [0.58, 0.71]	0.64 [0.58, 0.69]	0.65 [0.58, 0.71]	0.62 [0.56, 0.76]
GP21	1.28 [1.11, 1.45]	1.34 [1.17, 1.55]	1.28 [1.12, 1.46]	1.40 [1.13, 1.58]	1.28 [1.11, 1.46]	1.40 [1.19, 1.62]
GP22	6.09 [5.32, 7.06]	5.13 [4.57, 5.79]	6.01 [5.26, 6.99]	5.38 [4.87, 5.81]	5.99 [5.26, 6.97]	5.22 [4.67, 6.12]
GP23	2.75 [2.28, 3.45]	2.87 [2.25, 3.68]	2.74 [2.27, 3.45]	3.10 [2.36, 3.64]	2.75 [2.27, 3.44]	2.79 [2.29, 3.81]
GP24	4.49 [4.02, 4.98]	4.35 [3.56, 4.84]	4.48 [4.01, 4.98]	4.03 [3.44, 4.68]	4.48 [3.99, 4.97]	4.48 [3.77, 4.85]
GP25	31.55 [29.30, 33.55]	31.17 [28.64, 33.91]	31.51 [29.20, 33.53]	32.32 [30.33, 33.97]	31.52 [29.27, 33.58]	31.17 [27.40, 33.40]
GP26	1.41 [1.23, 1.60]	1.40 [1.23, 1.57]	1.41 [1.23, 1.60]	1.46 [1.34, 1.57]	1.41 [1.23, 1.60]	1.36 [1.19, 1.58]
GP27	5.43 [4.71, 6.17]	4.94 [4.27, 5.71]	5.41 [4.67, 6.16]	5.11 [4.46, 5.65]	5.40 [4.67, 6.16]	4.97 [4.26, 5.77]
GP28	3.17 [2.68, 3.74]	3.13 [2.62, 4.01]	3.16 [2.67, 3.76]	3.22 [2.81, 3.93]	3.16 [2.67, 3.76]	3.43 [2.75, 4.44]
GP29	1.83 [1.55, 2.06]	1.81 [1.42, 2.13]	1.82 [1.54, 2.07]	1.75 [1.39, 1.96]	1.81 [1.54, 2.07]	1.85 [1.46, 2.13]
GP30	0.30 [0.24, 0.36]	0.29 [0.23, 0.37]	0.30 [0.24, 0.36]	0.29 [0.23, 0.34]	0.30 [0.24, 0.36]	0.29 [0.23, 0.37]
GP31	1.06 [0.89, 1.23]	1.04 [0.89, 1.21]	1.05 [0.89, 1.23]	1.11 [0.93, 1.29]	1.06 [0.89, 1.23]	1.06 [0.91, 1.21]
GP32	0.61 [0.51, 0.73]	0.66 [0.53, 0.77]	0.62 [0.51, 0.73]	0.64 [0.54, 0.77]	0.62 [0.51, 0.73]	0.62 [0.51, 0.75]
GP33	0.96 [0.81, 1.13]	0.93 [0.76, 1.07]	0.96 [0.81, 1.13]	0.83 [0.77, 1.00]	0.95 [0.81, 1.12]	1.00 [0.73, 1.11]
GP34	6.14 [5.07, 7.31]	5.73 [4.49, 7.04]	6.11 [5.03, 7.30]	5.95 [4.93, 6.97]	6.11 [5.04, 7.30]	5.69 [4.52, 7.05]
GP35	0.44 [0.36, 0.54]	0.42 [0.33, 0.54]	0.44 [0.36, 0.54]	0.41 [0.32, 0.53]	0.44 [0.36, 0.54]	0.41 [0.35, 0.51]
GP36	0.57 [0.46, 0.72]	0.57 [0.43, 0.69]	0.57 [0.46, 0.72]	0.58 [0.42, 0.67]	0.57 [0.46, 0.72]	0.59 [0.46, 0.71]
GP37	1.76 [1.43, 2.13]	1.81 [1.53, 2.21]	1.75 [1.43, 2.14]	1.92 [1.73, 2.40]	1.76 [1.43, 2.15]	1.80 [1.52, 2.12]
GP38	3.24 [2.31, 4.20]	3.19 [2.42, 4.45]	3.24 [2.32, 4.20]	3.30 [2.49, 4.50]	3.24 [2.32, 4.21]	3.25 [2.55, 4.39]
GP39	0.45 [0.38, 0.53]	0.47 [0.39, 0.55]	0.45 [0.38, 0.54]	0.48 [0.41, 0.56]	0.45 [0.38, 0.54]	0.48 [0.39, 0.54]
GP40	0.41 [0.31, 0.53]	0.43 [0.33, 0.58]	0.41 [0.32, 0.53]	0.45 [0.34, 0.59]	0.41 [0.32, 0.53]	0.41 [0.32, 0.58]
GP41	0.45 [0.38, 0.53]	0.48 [0.41, 0.59]	0.45 [0.38, 0.53]	0.48 [0.44, 0.55]	0.45 [0.38, 0.53]	0.48 [0.38, 0.62]
GP42	0.28 [0.22, 0.35]	0.31 [0.21, 0.38]	0.28 [0.22, 0.36]	0.29 [0.19, 0.36]	0.28 [0.22, 0.36]	0.29 [0.20, 0.38]
GP43	0.42 [0.35, 0.49]	0.43 [0.37, 0.55]	0.42 [0.35, 0.49]	0.43 [0.37, 0.53]	0.42 [0.35, 0.49]	0.46 [0.36, 0.58]
GP44	0.22 [0.18, 0.27]	0.24 [0.19, 0.31]	0.22 [0.18, 0.27]	0.23 [0.18, 0.26]	0.22 [0.18, 0.27]	0.26 [0.19, 0.33]
GP45	0.25 [0.19, 0.31]	0.27 [0.21, 0.35]	0.25 [0.19, 0.31]	0.26 [0.20, 0.35]	0.25 [0.19, 0.31]	0.27 [0.20, 0.36]
GP46	0.16 [0.12, 0.22]	0.19 [0.13, 0.26]	0.16 [0.12, 0.22]	0.19 [0.13, 0.25]	0.16 [0.12, 0.22]	0.19 [0.14, 0.26]

Data are medians and interquartile ranges (in brackets). The raw data are represented (in percentage of abundance), although for statistical comparisons they were previously transformed into centered log-ratios. P values were obtained using the Mann-Whitney test, and the Benjamini-Yekutieli correction was applied to control the false discovery rate. The abundance of GPs highlighted in red is significantly higher than the abundance of the same GPs in the respective reference group. The abundance of GPs highlighted in mauve is significantly lower than the abundance of the same GPs in the respective reference group.

Cardiov., cardiovascular; GP, glycome peak.
